# Strengthening supportive supervision: A case study of the Expanded Programme on Immunization in Sindh, Pakistan

**DOI:** 10.7189/jogh.11.06004

**Published:** 2021-10-09

**Authors:** Sana Tanzil, Yasmeen Suleman, DureSamin Akram, Lubna Baig, Faraz Khalid

**Affiliations:** 1 APPNA Institute of Public Health, Jinnah Sindh Medical University, Karachi, Sindh, Pakistan; 2 Health Education and Literacy Program (HELP), DHA Phase V, Karachi, Sindh, Pakistan; 3 Consultant, UNICEF Pakistan, Islamabad, Pakistan

## Abstract

**Background:**

Sindh, one of the provinces of Pakistan, has been showing a consistently low coverage of immunization. Evidence supports the independent role of supportive supervision in improving the performance of immunization services. However, there is a dearth of evidence regarding the implementation of supportive supervision by the Expanded Programme on Immunization (EPI) Sindh and factors affecting its implementation in the local context.

**Methods:**

An exploratory case study was conducted in two districts of the province, Sindh ie, Hyderabad and Thatta. In total, 11 key informant interviews (KII) and 5 focus group discussions (FGDs) were conducted to obtain perspectives of various stakeholders of EPI, who play different roles in implementation of supportive supervision. Observations of EPI Checklist and review of current EPI policy and Module-4 of ‘Mid-Level Manager training’ by World Health Organization (WHO) for supportive supervision was also conducted.

**Results:**

This study reveals a lack of clarity regarding the potential role of supportive supervision amongst the stakeholders. Lack of human resources, limited competencies of supervisors, lack of specific training of concerned personnel and feedback mechanisms are major bottlenecks affecting the implementation of supportive supervision by EPI Sindh.

**Conclusions:**

The study concludes that supportive supervision is severely affected by challenges such as a lack of comprehensive EPI policy, unavailability of selection criteria for supervisors, training guidelines and proper logistic support to supervisors. There is a lack of training, motivation, and accountability amongst EPI personnel.

Expanded Programme on Immunization (EPI) is a nationwide government programme providing immunization services in Pakistan. EPI was launched in 1978, initially providing protective antigens against tuberculosis, poliomyelitis, diphtheria, pertussis and tetanus. Gradually over the years, vaccines against Measles, Hepatitis B, Hemophilus Influenzae type b (Hib), Pneumococcal vaccine, and Rotavirus as well as tetanus for pregnant women were added [1]. The programme provides both facility-based and outreach services [1]. The basic aim of the programme is to support provinces and districts in providing high-quality immunization services to prevent mortality, morbidity and disability due to vaccine-preventable diseases (VPDs) and contribute to the strengthening of national health systems [1,2].

Although, persistent efforts by EPI have led to some improvement in the vaccination coverage of children age 12-23 months, from 29% in 2012 to 35% in 2014 [2,3]. However, the vaccination rate of fully immunized children remains below the EPI target. Similarly, measles vaccine coverage has increased over the past two years from 45% in 2012 to 53% in 2014; still far below the EPI target of 90%. Ensuring effective management of available resources and optimum performance of current human resource at various levels are crucial challenges for EPI, especially in Sindh and Baluchistan which are lagging behind other provinces of Pakistan in terms of immunization coverage [2,3].

Weak supervision and monitoring are among the key factors causing sub-optimal performance in EPI [4]. An inadequate number of supervisors and lack of their skills to provide supervision are major factors that can affect accountability towards the delivery of quality immunization services [4,5]. In the current EPI administration, the district superintendent of vaccination (DSV) and *taluka* (sub-district) superintendent of vaccination (TSV) are responsible for the supervision of immunization services [6]. Additionally, district health officers (DHO), additional district health officers (ADHO) and District EPI focal persons are responsible for supervising the programme at their respective levels. At the provincial level programme managers and deputy programme managers are responsible [6]. However, monitoring is done infrequently, and/or the monitoring personnel lack the capacity required to perform the task [6].

*Supportive supervision* is a process of helping health staff to improve their knowledge, skills and work performance in a respectful and non-authoritarian way [7]. It focuses on monitoring performance towards goals using data for decision-making and depends on a regular follow-up to ensure effective implementation of tasks. Supportive supervision can have independent positive effects on immunization programme indicators [8]. A study conducted in India found positive effects of supportive supervision on the knowledge and practices of routine immunization service providers [9].

In 2002, World Health Organization (WHO) and its partners developed the ‘Reaching Every District’ (RED) strategy for increasing and sustaining high levels of routine immunization. Supportive supervision is a component of the RED strategy [10]. An evaluation of the RED strategy conducted in five African countries showed that it contributed significantly to strengthening immunization systems and improving the delivery of vaccines [10]. The strategy has been adopted in Pakistan and mentioned in the current National EPI policy 2015.

However, there was a dearth of relevant evidence to determine and address the factors influencing the successful implementation of supportive supervision. The study aims to obtain a holistic view of the current state of implementation of supportive supervision for routine immunization services in Sindh. It also aims to document barriers and facilitators to the process and thereby establishing the scientific evidence required to improve its implementation.

## METHODS

### Study design and settings

A case study of supportive supervision by EPI Sindh was designed and conducted using an exploratory qualitative research approach. This study was conducted in two districts of the province which have completed mid-level manager (MLM) training and are implementing supportive supervision, ie, Hyderabad and Thatta from August to October 2017. District, Hyderabad majorly represents the urban population with extended peri-urban or semi-rural communities. However, district Thatta mainly represents rural Sindh with a geographically dispersed population with limited resources. The two districts were chosen purposefully to obtain a representative view of the situation in Sindh regarding supportive supervision. This also provided an opportunity to identify and appreciate any possible differences in the implementation of supportive supervision in urban and rural settings of Sindh. However, to obtain a holistic view of the situation this study explored the implementation of supportive supervision at multilevel by using variable sources of information which included a review of the existing “National EPI Policy and Strategic Guidelines, Pakistan”, review of “WHO MLM Training -Module:4”. Qualitative assessment of the implementation of supportive supervision with FGDs and KIIs.

### Study participants

The study identified various stakeholders of EPI Sindh as study participants. It included stakeholders who are key players and responsible for the implementation of supportive supervision and provision of routine immunization services in Hyderabad and Thatta. These include TSV, DSV, District Health Officer (DHO) and vaccinators. The project Director of EPI Sindh and representatives from UNICEF and the WHO were also included in the study due to their influential role in suggesting policy prescriptions and deciding EPI direction and strategies.

### Sampling

The study participants were selected purposively. The data was collected using qualitative techniques ie, FGDs and Key Informant Interviews (KIIs).

In total 11 KIIs were conducted to obtain perspectives of various stakeholders of EPI, Sindh who play different roles in the implementation of supportive supervision. A summary of the demographic detail of the study participants and data collection methods is presented in **Table 1****.**

**Table 1:** Overview of study sample and data collection from various stakeholders of EPI, Sindh

S. No	Study participants	Representative group of stakeholder	Data collection technique	Place of interview/Discussion
1	DHO	DHMT	KII	Hyderabad
2	DSV	EPI	KII	Hyderabad
3	TSV	EPI	KII	Hyderabad
4	Vaccinator	EPI	KII	Hyderabad
5	Vaccinators	EPI	FGD	Hyderabad
6	Vaccinators	EPI	FGD	Hyderabad
7	Immunization Officer, UNICEF	Policy maker and technical partners	KII	Karachi
8	Head of Office EPI Sindh, WHO	Policy maker and technical partners	KII	Karachi
9	PD-EPI	EPI	KII	Karachi
10	DHO	DHMT	KII	Thatta
11	ADHO	DHMT	KII	Thatta
12	DSV	EPI	KII	Thatta
13	Vaccinator	EPI	KII	Thatta
14	TSVs	EPI	FGD	Thatta
15	Vaccinators	EPI	FGD	Thatta
16	Vaccinators	EPI	FGD	Thatta

EPI – expanded program for immunization, DHO – district health officer, ADHO – assistant district health officer, DHMT – district health management team, FGD – focus group discussions, KII – key informant interviews, TSV – Tehsil/Taluka supervisor vaccination, VPD – vaccine preventable diseases

**Figure 1:**
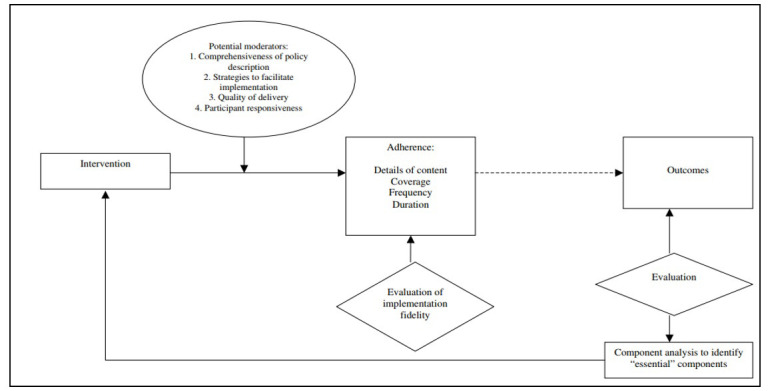
Conceptual framework to measure implementation fidelity.

**Figure 2:**
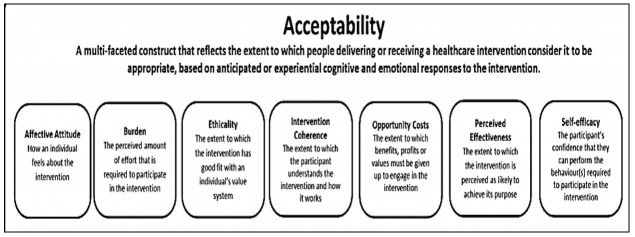
Conceptual framework to measure implementation acceptability.

**Data collection:** The qualitative data were collected through FGDs and KIIs. The interview guidelines comprised of a list of open-ended questions, probes and instructions for the interviewer to administer the KIIs and FGDs. Guidelines were developed based on the conceptual frameworks of fidelity and acceptability adapted from previously conducted studies [11,12]. Fidelity was defined using four major constructs which included adherence, frequency, quality and responsiveness (**Figure 1**). However, acceptability was described using specific constructs such as value or utility and conformability and suitability [11,12]. (**Figure 2**).

An observation checklist was also developed to observe the quality and coverage of information reported by supervisors (including completeness of reported information and data quality). The data collection tools were tested before the start of actual data collection. Data was mainly collected by qualified and experienced researchers including Co-Investigators. Each data collection team comprised three personnel including an interviewer, note-taker and observer. Video and audio recordings of each FGD and KI interviews were done using a camera and voice recorder after taking informed consent from study participants.

The observation of EPI records and reports of supportive supervision was also conducted. By verifying EPI records for checklists of supportive supervision in both study districts. The observation was conducted for fixed EPI facilities as well as outreach immunization services with the help of a specifically designed checklist.

Review of the existing EPI Policy and WHO training manual was conducted independently by two different researchers. The purpose of the EPI policy review was to identify any documented policy and related strategy for supportive supervision of immunization services. Similarly, the WHO training manual for the Training for mid-level managers (MLM), Module: 4 was also reviewed [7]. This particular module of MLM training is specifically designed to provide training on supportive supervision. Hence, it was independently reviewed by two researchers in detail to identify the content of training for supportive supervision provided to district health management teams or mid-level managers. The observations of both the researchers who reviewed EPI policy and the MLM manual were noted individually by each researcher. Conclusions were drawn with the agreement between the two researchers after discussion.

Furthermore, one monthly meeting was attended for each study district to obtain a holistic overview of the feedback mechanisms and response and approach of district health authorities towards reacted challenges. The meeting was attended by the same researcher who conducted observations of the checklist. The proceedings of the meeting were observed and recorded in writing and by a video camera.

### Data analysis

The data collected through FGDs and KIIs was transcribed by the trained personnel. The transcribed information was translated by a language expert into English. The transcripts were analyzed using thematic content analysis. Both “manifest content” and “latent content” of the text were analyzed by two different researchers. Finally, two researchers independently reviewed the codes and identified sub-categories and categories. The latent content was expressed in sub-themes and themes after systematically analyzing the commonalities, variations and disagreements of the three researchers. Information coming through the checklist, observation notes for EPI monthly meeting and document review including policy analysis supplemented the information coming through FGDs and KIIs. All the data analysis process was continuously supervised by a third researcher with extensive experience and skills in qualitative research. The third researcher analyzed the data and provided input regarding emerging themes and subthemes to decide on disagreements between the two researchers. The information from the observation checklist was entered into SPSS and frequencies were calculated for completion of the content of the EPI checklist. The triangulation of information coming through three different methods of data collection was done to enhance the validity and credibility of study findings.

### Ethical considerations

The ethical approval for this study was obtained by the Institutional Review Board (IRB) of Health Services Academy, Islamabad before the study was initiated. Approval and letter of support were obtained from Project Director EPI Sindh and submitted to the IRB), donor agencies and DHO offices of two included districts. Written informed consent was obtained from all study participants prior to each FGD and KII. At the end of the project, EPI Sindh and other relevant stakeholders were provided with specific recommendations to strengthened supportive supervision in Sindh.

## RESULTS

### Review of EPI policy regarding supportive supervision of immunization services

The existing National EPI policy and strategic guidelines Pakistan 2015 is a key guiding document for the implementation of Immunization services. The policy was developed by Federal EPI with technical assistance from WHO and UNICEF and participation of other stakeholders including Provincial EPI cells and National and International non-government organizations (NGO). The Policy mentions the RED strategy and has brief and ambitious guidelines for supervision and monitoring but It does not give clear directions to adapt and implement supportive supervision. It doesn’t mention any specific implementation strategy, nor does it assigns specific role and responsibilities of various cadres of EPI cadres for supportive supervision and program implementation.

### Review of WHO training module for supportive supervision

The WHO training module for supportive supervision is part of Mid-Level Managers (MLM) training and is available only in English. It directly addresses the training needs of EPI personnel and provides a comprehensive description of the role of supportive supervision as well as a structured guideline about the role and activities of the EPI supervisors.

The module deals with the storage, cold chain, stacking and stock management but, has very little information regarding the actual administration of vaccines, filling, out of cards and counseling. The checklist provides sufficient information about administrative aspects including monitoring of facilities but guidance regarding dealing with clients, counseling by vaccinators, recording of AEFIS and their management is missing. Additionally, indicators related to the supervision of supervisors are not mentioned.

### Qualitative assessment with FGDs and KIIs

#### Perceptions about supportive supervision

The respondents had varied perceptions about supportive supervision of immunization services. A respondent from the vaccinator group said *“Monitoring is done just to keep an eye that nothing is stolen”.* According to one of the policymakers, *“Supportive supervision means you must be technically strong”.* Most respondents perceived supportive supervision and monitoring as the same. One of the supervisors said, “*There is not much difference, both are almost the same”*.

The respondents mainly from the District Health Management Team (DHMT) and policymakers, who perceived supportive supervision and monitoring as two different activities predominantly believed supervision to be better than monitoring. Provision of support to vaccinators to help them solve issues related to performance and service quality was seen as the main purpose of supportive supervision. Optimal utilization of the workforce, an increased sense of responsibility and improved performance of immunization services were mentioned as the major outcomes in support of the effectiveness of supportive supervision. As one policymaker said, *“ It contributes to the betterment of practices”.* Likewise, a respondent from the DHMT said, *“Supportive supervision improves the performance of immunization services”*

The predominant perceptions of vaccinators was that the quality of current implementation and effectiveness of supportive supervision is poor, there is a lack of trained supervisors, a dearth of field visits by supervisors and scolding of vaccinators by supervisors. One vaccinator said *“Vaccinators do not expect much from the supervisors because they have not received any training”* another said, *“Instead of motivating vaccinators they are being scolded and pressurized”.*

### Components of supportive supervision implemented by EPI, Sindh

Respondents identified 1) visiting the vaccinators at health facilities and in the community outreach, 2) verification of immunization records and filling of EPI checklist for supportive supervision, 3) provision of support to vaccinators and 4) feedback and reward mechanism, as the most important components of the implementation of supportive supervision.

All the respondents confirmed that supervisory visits are conducted by all four tiers of EPI supervisors ie, TSV, DSV, District focal persons EPI and DHO/ADHO. More than three fourth of the vaccinators and supervisors mentioned more frequent visits by TSV than by DSV, DHO and ADHO. The majority of respondents mentioned monthly supervisory visits by TSV. Others said that the frequency of visits at a facility varies with its performance, with a minimum one monthly visit.

Almost all the respondents mentioned checking immunization records as an integral part of supervision. The supervisors verify the records of immunization coverage from the record registers at the facilities and conduct a coverage cluster survey to assess immunization coverage in outreach. As one of the vaccinators said, *“They check register, visit the field and take cluster too”.* All cadres of supervisors use the same EPI supervisory checklist.

The TSVs and DSVs provide technical support to the vaccinators. This includes training of vaccinators to fill gaps in knowledge and skills, support and guidance to improve immunization coverage. One to two vaccinators at each study site mentioned that if and when required, supervisors help them in performing vaccination too; as one of them said, *“If there is a big crowd they help us in vaccination, as TSVs are from us.”*

Only a few vaccinators and supervisors reported the existence of any rewards mechanisms.. According to them, feedback is provided to the bad as well as good performers. Complaint to DHO, issuance of a letter by the DHO asking for an explanation for poor performance and delay or discontinuation in salary were mentioned as the major punishment mechanisms for poorly performing vaccinators. Good performance is rewarded usually by applause in the monthly review meeting, occasionally awarding a certificate or a financial incentive. One of the vaccinators said, “*Fuel money is performance-based; if vaccinator is doing well he gets 400 in salary as fuel money”.* The respondents mentioned the need to improve existing feedback and reward mechanisms.

### Competency of supervisors involved in supportive supervision of EPI services

Almost every respondent reported a severe lack of skills and training of concerned personnel involved in supportive supervision. As stated by most of the respondents, job seniority and capability to work as a supervisor are major criteria practiced by EPI for promoting a vaccinator to the supervisor. Different levels of qualifications for selection as a supervisor in EPI, Sindh ie, 10 and 12 grades of schooling were reported by supervisors from DHMT and EPI respectively. According to one of the policymakers, the supervisors are selected from the same area where they are deputed. Two of the vaccinators mentioned the existence of a practice of getting a promotion to the post of supervisor by paying a bribe to the concerned clerk.

### Training of supervisors

Respondents from DHMT stated that DHO trains supervisors in supportive supervision. Giving vaccination, filling supervisor's checklist, and interpersonal communication were mentioned as the main topics included in this training. The DHO also updates them on the new developments in EPI during monthly review meetings. They also mentioned that EPI conducts training of supervisors. Contrary to respondents from DHMT, the supervisors pointed out that proper training is not given to them. DHOs provide only informal training. Further, all of them emphasized the need for specific and formal training. As one of the TSV said, *“There is a lot of difference in learning and getting trained properly”.* All the supervisors pointed out to non-availability of any written protocol or guideline for supervisors to conduct supervisory visits. The need for training of supervisors in interpersonal communication skills was also emphasized by DHOs.

### Roles and responsibilities

According to almost all the respondents, the predominant role of DHMT, particularly DHO is the administration of vertical programs including EPI. DHMT mainly supervises the overall process of supportive supervision. DHO is responsible at the district level and manages EPI services as well as provides support to vaccinators and EPI supervisors.

According to DSVs and TSVs, DHMT maintains accountability among all EPI personnel. DHO performs field visits and develops his own report and verifies the supervisor’s report with his report in the monthly meeting. The predominant responsibility of TSV and DSV as stated by the majority of respondents is to assess whether an immunization service facility is performing well and provision of support needed to correct any gaps identified in the performance of vaccinators. All supervisors and few vaccinators among respondents also mentioned that TSV reports to DSV weekly while the DSVs also visit each facility.

Provision of technical support help in micro-planning and provision of fuel for outreach services and evaluation of EPI performance in terms of immunization coverage as a third party were mentioned as the predominant roles of NGOs in Sindh.

### Factors affecting the implementation of supportive supervision

One of the major concerns repeatedly mentioned by most of the respondents was the shortage of human resource for performing supportive supervision and their inappropriate geographical distribution. One of the vaccinators said, *“In five Union Councils (UCs) there is one TSV; there should be one TSV for two UCs”.* Moreover, lack of essential skills among supervisors themselves was another most reported concern as mentioned by stakeholders from all levels. As one of the vaccinators said; *“DSVs themselves haven’t received any training yet”*.

According to some supervisors, their requests to attend MLM training conducted by EPI were not entertained. As one of the supervisors raised his concern against lack of training and mentioned *“MLM trainings were conducted in April and it was clearly written in the letter that TSV and DSV are not allowed to attend training”.* A few EPI supervisors mentioned that supervision, especially in far-flung areas, is hampered by inadequate transport facilities. They identified this as a major barrier and highlighted that the support provided for fuel is inadequate and not regular. Nevertheless, almost all supervisors blamed the increased workload and multiple responsibilities as a factor affecting supervisory visits by respective authorities such as involvement in polio campaigns. As one district official said, *“If you give them multiple jobs then they are unable to fulfill basic job requirements.”*

The majority of the respondents stated that lack of monetary and non-monetary incentives affects the performance of EPI supervisors and stressed on the need for regular recognition and appreciation for staff motivation. On the other hand, a representative from developmental partners mentioned that:*“Monitoring of that monitor is not done”.* Another respondent stated, *“The main thing is, there is no accountability; nobody is held accountable.” However, FGDs and KIIs with vaccinators also* identified the political influence as one of the reasons for lack of commitment and poor performance among EPI supervisors; one of them said, *“Political influences are there in poor performance”.* Another said, “political issues; yes, it is everywhere”.

### Recommendations by the respondents to improve supportive supervision

Almost all the respondents stressed on the need for appropriate placement of personnel for supportive supervision and the need for capacity building of DSVs and TSVs. Supervisors and the development partners recommended the provision of appropriate transport facilities, especially in far-flung areas. Strengthening of coordination mechanism was also recommended by one of the development partners; *“EPI Sindh has better coordination, but it needs further improvement”.*

### Observation of completeness and quality of information collected by the supervisors using EPI checklist

**Table 2** summarizes the frequency by which selected items on the EPI checklist were correctly filled by supervisors. It points to poor quality of data collection. However, it was comparatively better in Hyderabad than in Thatta.

**Table 2:** Observation of completeness of information filled by supervisor on EPI checklist for supervision

	Hyderabad (n = 5)	Thatta (n = 5)
**Items correctly filled by supervisor - EPI checklist**	Hyderabad	
Date of visit	4	3
District name	5	3
Taluka number	5	3
Union council number	4	3
Health facility type	2	1
EPI manpower number at facility	3	4
Shortage of vaccine	5	3
Information about vaccine storage	4	3
Availability and use of safety boxes	5	2
Working cold chain equipment available	5	4
Display of updated vaccination monitoring chart	3	3
Monthly outreach activity plan duly approved and signed by facility in charge	3	4
Updated list of defaulters available with vaccinator	0	0

EPI – expanded program for immunization

## DISCUSSION

This study provides a comprehensive overview of the current status of the implementation of supportive supervision in Sindh. Though the current EPI policy mentions supervision and monitoring of immunization services, it does not provide a clear description of the aims and objectives of supportive supervision and lacks specific direction. Based on this policy, training on supportive supervision was introduced for EPI managers responsible for monitoring. The WHO training manual for Mid-Level Manager-Module:4 was used for this purpose. It provides a comprehensive and detailed description and structured guideline about the role and activities of the EPI supervisors; the training is limited to DHOs and ADHOs. Frontline supervisors ie, DSVs and TSVs are not being trained. Further, the training module is available only in English. Translation of the module in the local language can prove helpful in the training of frontline supervisors and improving its implementation fidelity.

This study identified a considerable lack of clarity among EPI stakeholders regarding the actual role and scope of supportive supervision in improving immunization services. Many of the EPI supervisors including DSV and TSVs could not differentiate between monitoring and supportive supervision and perceived supportive supervision as an activity to police the vaccinators. This reflects prevailing misunderstandings regarding the role of supportive supervision in immunization, which may affect implementation. The misinterpretation of objectives of supervision is consistent with Bradley’s study, which found that supervision was perceived by immunization personnel as inspection, primarily to find the faults and weaknesses among health care service providers [13]. Despite this supportive supervision has good acceptability among all EPI cadres. They appreciated its usefulness in enhancing the quality of immunization services and ensuring accountability.

The fidelity of supportive supervision varies widely across the districts and has been severely affected by program-related challenges. These include lack of skilled personnel, inappropriate placement, poor logistic support and a lack of clarity on the role and responsibilities of supervisors.

The study identified an immense need for the development of specific skills required for effective implementation of supportive supervision. These include interpersonal communication skills, technical capacity and updates on new developments. Moreover, a practice of regular refresher training on supportive supervision needs to be adapted to strengthen its implementation in the long run. This finding is consistent with the study conducted by Meena and colleagues [9].

This study found that the supervisor is expected to perform various activities in his supervisory visit including assessing the vaccinator`s technique of giving the vaccine and his/her counseling skills, evaluation of vaccine storage equipment and practices and verification of coverage records. This requires sound knowledge and specific technical and managerial skills and specific competencies among EPI supervisors. Monthly review meetings conducted in each district by DHO were identified as the main platform for assessing the performance of vaccinators as well as their supervisors and provide feedback. Most of the respondents were satisfied with the conduct of monthly meetings and acknowledged these meetings as a learning opportunity. The importance of such review meetings is supported by Shimp and colleagues; who analyzed the data from 200 review meetings of immunization services and concluded that review meetings helped in building the technical capacity of immunization staff who attended those meetings [14].

The study also identified quality issues in filling EPI checklists by TSVs and DSVs for the observed EPI facilities. This further validates the reported lack of competence and need for training as well as a low-performance motivation among EPI supervisors in Sindh. Lack of necessary skills or low self-efficacy to perform assigned tasks may result in low performance and may work as a barrier. A study conducted in Benin concluded that lack of motivation, poor coordination and inadequate management skills were major barriers to the supervision of services [15]. Political interference was identified as another factor influencing supportive supervision as was the poor performance of the Programme itself hindering effective implementation of supportive supervision. Supportive supervision cannot improve the quality of immunization services until system issues are addressed. Availability of essential logistics, supply chain management, timely indenting, and financial resources, could complement the supportive supervision strategy in improving immunization service delivery [16].

The major strength of this study was, that it gathered data from multiple cadres of EPI Sindh and other stakeholders from urban as well as rural settings. The detailed document review of EPI policy and supervisor training module enhanced understanding of the researchers about the process of supportive supervision and the role of the supervisor. Participation in monthly EPI district meetings helped in recognizing the actual practices and feedback mechanisms in place and their intensity. The study has used methodological triangulation by using FGDs, KIIs, desk review of documented observation as methods of data collection. This enhances the in-depth understanding of the issue under study and increases the trustworthiness of the study findings. The study tools were designed using specifically identified frameworks and pre-tested for lingual and cognitive validity, hence the enhanced quality of information and credibility and dependability of the study findings. Nevertheless, this study can serve as a milestone; to reform the existing EPI system which may include incorporating supportive supervision of EPI services as a priority agenda in the existing policy document. Moreover, this study brings attention to the restructuring of recruitment or selection criteria of EPI supervisors, training structure for supportive supervision as well as effective management of resources to ensure quality service delivery by all cadres in the existing system.

The study has a few limitations. First, the KIIs with TSVs were not completed in one study district due to the limited number of TSVs. This may have failed to explore any unique perspectives from those who may have left due to context-related factors such as suboptimal working conditions related to lack of standard protocols. Second, the selection of facilities for observation was based on checklists provided by DHMT. Hence, the possibility of biased selection of facilities with more regular and adequate supervision cannot be ruled out.

## CONCLUSIONS

The study concludes that despite ambiguities regarding the actual role of supervisor and supportive supervision, supportive supervision itself has good acceptability among all EPI personnel. The fidelity of supportive supervision varies widely across the districts. It is severely affected by program-related challenges such as lack of comprehensive policy and unavailability of pertinent training guidelines. The study identified that supervisors had a lack of motivation due to the unavailability of regular performance-based incentives and lack of accountability. Improved feedback mechanisms need to be developed to enhance the accountability of EPI stakeholders, including supervisors. The perception of supervision has to be revised from policing to support. The study findings suggest extensive changes and developments at multiple levels to make the accountability mechanism effective and to ensure its acceptability and fidelity at all levels in EPI Program. A revised EPI policy with sufficient description of each stakeholder and specific strategy may help the program and increase the acceptability of supportive supervision and of supervisors.
